# Hybrid Skeleton-Based Motion Templates for Cross-View and Appearance-Robust Gait Recognition

**DOI:** 10.3390/jimaging12010032

**Published:** 2026-01-07

**Authors:** João Ferreira Nunes, Pedro Miguel Moreira, João Manuel R. S. Tavares

**Affiliations:** 1 Escola Superior de Tecnologia e Gestão, Instituto Politécnico de Viana do Castelo, Avenida do Atlântico, 4900-348 Viana do Castelo, Portugal; 2 ADiT-Lab, Instituto Politécnico de Viana do Castelo, 4900-347 Viana do Castelo, Portugal; pmoreira@estg.ipvc.pt; 3 Instituto de Ciência e Inovação em Engenharia Mecânica e Engenharia Industrial, Departamento de Engenharia Mecânica, Faculdade de Engenharia, Universidade do Porto, 4200-465 Porto, Portugal; tavares@fe.up.pt

**Keywords:** gait recognition, skeleton-based representation, motion templates, human pose estimation, Gait Skeleton Image (GSI), covariate robustness, viewpoint variation, appearance variation, CASIA-B, GRIDDS

## Abstract

Gait recognition methods based on silhouette templates, such as the Gait Energy Image (GEI), achieve high accuracy under controlled conditions but often degrade when appearance varies due to viewpoint, clothing, or carried objects. In contrast, skeleton-based approaches provide interpretable motion cues but remain sensitive to pose-estimation noise. This work proposes two compact 2D skeletal descriptors—Gait Skeleton Images (GSIs)—that encode 3D joint trajectories into line-based and joint-based static templates compatible with standard 2D CNN architectures. A unified processing pipeline is introduced, including skeletal topology normalization, rigid view alignment, orthographic projection, and pixel-level rendering. Core design factors are analyzed on the GRIDDS dataset, where depth-based 3D coordinates provide stable ground truth for evaluating structural choices and rendering parameters. An extensive evaluation is then conducted on the widely used CASIA-B dataset, using 3D coordinates estimated via human pose estimation, to assess robustness under viewpoint, clothing, and carrying covariates. Results show that although GEIs achieve the highest same-view accuracy, GSI variants exhibit reduced degradation under appearance changes and demonstrate greater stability under severe cross-view conditions. These findings indicate that compact skeletal templates can complement appearance-based descriptors and may benefit further from continued advances in 3D human pose estimation.

## 1. Introduction

Human gait analysis has long been recognized as a powerful non-invasive tool for understanding locomotion, supporting clinical diagnosis, and enabling biometric recognition. In the past decade, significant advances in sensing technologies and computer vision have led to substantial progress in extracting meaningful information from human motion, even in unconstrained environments. Concurrently, the advent of deep learning has precipitated a paradigm shift from handcrafted descriptors to data-driven representations capable of capturing subtle spatiotemporal patterns directly from raw or pre-processed sensor data.

Appearance-based representations derived from binary silhouettes remain highly attractive for gait analysis due to their robustness, low computational cost, and independence from background clutter or clothing textures. Among these, the Gait Energy Image (GEI) and its variants have established themselves as influential baselines. Nevertheless, silhouette-averaged descriptors inherently lose fine-grained temporal cues and may suffer performance degradation under significant viewpoint changes or covariate variations.

In parallel, skeleton-based methods, whether derived from RGB-D sensors or extracted from RGB video via pose-estimation networks, have gained substantial attention. These representations benefit from an interpretable structural encoding of human motion and can capture dynamics that silhouettes alone cannot fully describe. However, they may exhibit sensitivity to keypoint localization errors, occlusions, and variability introduced by different pose-estimation pipelines. Rather than viewing appearance- and skeleton-based approaches as competing paradigms, recent perspectives suggest that they can be complementary, capturing distinct but mutually reinforcing aspects of human gait.

Motivated by this complementarity, the present work proposes two variants of *Gait Skeleton Images* (GSI), which encode skeleton-derived motion into compact 2D templates following principles inspired by silhouette-based motion descriptors, while relying exclusively on skeletal information. The resulting templates are interpretable, compatible with standard 2D CNNs, and designed to (i) emphasize stable joint-trajectory structure, (ii) reduce sensitivity to appearance perturbations such as clothing and carried objects, and (iii) integrate into conventional CNN pipelines without recurrent models or multi-stream fusion.

This study provides a systematic evaluation of both GSI variants under standard gait-recognition benchmarks. Beyond benchmarking, we analyze key design aspects, including the trade-off between line-based and joint-based encodings and the influence of pixel-level rendering parameters. The results clarify how structural and spatial configuration choices affect discriminative behavior and robustness under viewpoint and appearance covariates.

### Contributions

This article introduces and evaluates two silhouette-inspired *Gait Skeleton Image* (GSI) representations that encode skeletal motion as compact 2D templates for CNN-based gait recognition. The main contributions are as follows:

- Two complementary GSI variants, a line-based encoding and a joint-based encoding, are formalized and characterized in terms of their structural design and spatiotemporal properties, providing skeletal templates that can be directly processed by standard 2D CNN architectures.
- A unified and reproducible processing pipeline is defined, transforming 3D joint coordinates from heterogeneous pose-estimation sources into a standardized 17-keypoint skeleton, followed by view and translation normalization, orthographic projection onto a canonical 2D plane, and GSI rendering in a normalized coordinate frame.
- Core design factors are systematically analyzed on the GRIDDS dataset, where 3D joint coordinates are captured directly from a depth sensor. This reliable acquisition setup provides stable skeletal data, enabling a controlled assessment of structural choices (line- vs. joint-based encodings) and pixel-level rendering parameters (line thickness and joint radius) under consistent lateral-view conditions.
- An extensive experimental evaluation is conducted on the widely used CASIA-B benchmark, where 3D joint coordinates are obtained via human pose estimation from RGB video. In this more challenging setting, which is subject to viewpoint changes, occlusions and appearance variations, the optimized GSI variants are compared against the Gait Energy Image (GEI) under identical CNN architectures, training protocols and evaluation conditions.
- The robustness of the proposed GSI representations is examined under key gait covariates (viewpoint, clothing, and carrying conditions), highlighting scenarios in which skeletal GSI templates offer more stable behavior than conventional appearance-based descriptors, while also quantifying their limitations in absolute same-view accuracy.

## 2. Related Work

Gait recognition has been widely explored as a distance-based biometric modality capable of identifying individuals from their walking patterns. Vision-based approaches are commonly grouped into appearance-based and skeleton-/model-based methods, which encode complementary identity cues and exhibit different failure modes under viewpoint and appearance covariates.

**Gait datasets and acquisition modalities.** The development and evaluation of gait recognition methods are strongly influenced by the characteristics of the datasets used, including sensing modality, viewpoint coverage, covariate diversity, and annotation quality. Early large-scale efforts focused on RGB-based silhouette datasets acquired under controlled conditions, while more recent benchmarks increasingly incorporate depth sensing and pose information. Firman [1] provided an early and comprehensive overview of RGB-D datasets, highlighting how depth data enables more reliable geometric and structural representations compared to purely appearance-based cues. Focusing specifically on gait analysis, Nunes et al. [2] conducted a systematic review of RGB-D gait datasets, analyzing acquisition setups, sensor configurations, subject diversity, and the availability of synchronized modalities such as RGB, depth, silhouettes, and skeletons.

**Appearance-based silhouettes and templates.** A dominant line of work derives descriptors from binary silhouettes, body contours, or motion templates. A seminal example is the Gait Energy Image (GEI) [3], obtained by averaging silhouette frames over a gait cycle to yield a compact spatial representation. Numerous variants have been proposed to address GEI limitations through part-based decompositions, temporal-gradient cues, or saliency weighting, maintaining popularity due to simplicity, low computational cost, and reduced dependence on texture [4,5]. However, the collapse of temporal information into static templates may limit the efficacy of appearance-based descriptors in scenarios involving covariate variations. These variations, such as viewpoint changes, clothing differences, and object carrying, are known to induce significant performance degradation.

**Graph-based skeleton representations.** With the maturation of depth sensors and human pose estimation, skeleton-based gait recognition has gained traction by focusing on joint kinematics and anatomical structure. Graph Convolutional Networks (GCNs) are a prominent paradigm, treating joints as nodes and bones as edges, and learning discriminative features from spatiotemporal skeleton sequences [6]. Representative approaches include GaitGraph and extensions based on spatiotemporal graph convolutions [7], as well as PoseMapGait, which integrates pose-related heatmaps and graph construction to learn invariant representations [8]. While effective, these methods typically require specialized graph operations and preserve the sequential nature of the data, thereby increasing architectural and computational complexity and making deployment more dependent on graph-processing toolchains.

**Skeleton-to-image transformations for CNNs.** A related direction involves the conversion of skeleton sequences into image-like representations, which are designed for standard CNN processing. Skepxels [9] arranges skeleton joint coordinates into two-dimensional grids and encodes dynamics through coordinate differences (e.g., velocity), producing multi-channel inputs that preserve temporal information. More recently, SkeletonGait [10] proposed skeleton maps that preserve the spatial layout of skeletal structure while remaining appearance-free, demonstrating competitive performance with CNN backbones. Furthermore, the potential of skeleton-derived image templates extends beyond mere identification, as evidenced by [11] which rendered 2D poses into GEI-like templates for gait-type/pathology classification under side-view acquisition. This indicates that pose-to-template mappings can support clinically relevant tasks.

**Hybrid fusion strategies and remaining gap.** To improve robustness under challenging covariates, hybrid strategies have combined silhouette and pose cues via multi-stream architectures, feature-level fusion, or shared embedding spaces [12,13]. Recent surveys highlight hybrid modalities as a promising direction for addressing viewpoint and appearance variation [14]. Despite this progress, there remains limited work on compact, unified 2D *motion-template* representations that (i) encode skeleton-derived spatiotemporal structure using principles traditionally associated with silhouette templates, (ii) incorporate explicit geometric normalization for viewpoint handling, and (iii) remain directly compatible with standard 2D CNN pipelines without recurrent modeling or graph-based operations.

**Positioning of the proposed GSI.** In this context, we introduce two *Gait Skeleton Image* (GSI) variants that encode skeleton-derived motion cues into compact 2D templates while excluding silhouette information. In contrast to graph-based models that operate on irregular skeletal topologies and process sequences explicitly, GSI converts 3D joint trajectories into regular-grid images suitable for conventional CNNs. Distinctively, GSI employs explicit 3D geometric normalization (view alignment and translation stabilization) prior to orthographic projection and GEI-inspired temporal aggregation over gait cycles, producing fixed-size templates from variable-length sequences. Furthermore, two rendering variants (line-based connectivity and joint-based markers) provide representational flexibility to emphasize either limb connectivity patterns or joint-trajectory structure. A two-stage evaluation is adopted: first, controlled design-factor analysis is conducted to select rendering parameters and encoding topology; second, robustness is assessed under viewpoint, clothing, and carrying covariates in a widely used benchmark setting.

## 3. Materials and Methods

**Figure 1:**
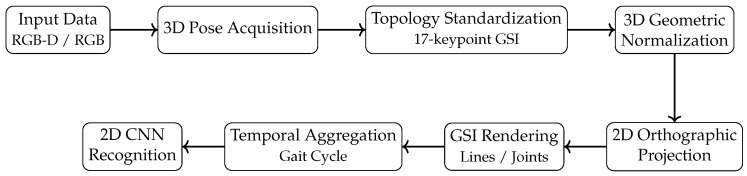
Overview of the proposed Gait Skeleton Image (GSI) processing pipeline.

This section describes the datasets, the skeletal preprocessing procedures, the proposed *Gait Skeleton Image* (GSI) variants, and the experimental pipeline used to evaluate the representations. Two publicly available datasets were used for complementary purposes: GRIDDS was used to analyze design factors related to pixel-level parameterization and structural encoding, while CASIA-B was used to evaluate robustness under viewpoint, clothing, and carrying covariates. Figure 1 provides a concise overview of the complete processing pipeline, illustrating the main processing stages from skeletal acquisition to CNN-based recognition.

### 3.1. Datasets

#### 3.1.1. GRIDDS Dataset

The GRIDDS dataset [15] offers a modality-rich benchmark designed for controlled gait analysis. All recordings were captured using a fixed Microsoft Kinect v2 sensor with subjects walking along two straight trajectories (left-to-right and right-to-left) under a stable lateral viewpoint. The dataset contains 35 participants, with each participant contributing 10 walking sequences, resulting in a total of 350 recordings. GRIDDS distributes synchronized RGB, depth, infrared frames, body-index silhouettes, and 2D and 3D skeletal joint coordinates. Kinect v2 provides 25 body joints per frame in camera coordinates, enabling accurate and consistent skeletal tracking throughout the sequences.

In this study, GRIDDS was used to analyze the design factors of the proposed GSI representations in the context of person recognition. The experiments systematically varied pixel-level parameters (line thickness and joint radius) and structural encoding choices (line-based versus joint-based topologies) to assess how these factors affect the discriminative capacity of the representations. The absence of confounding covariates such as clothing variation or carried objects, together with reliable depth-based tracking and stable lateral-view acquisition, makes GRIDDS particularly suitable for controlled and interpretable comparisons across different representation configurations.

#### 3.1.2. CASIA-B Dataset

The CASIA-B dataset [16] is one of the most widely used benchmarks for gait recognition. It contains 124 subjects recorded from 11 viewpoints (0–180° in 18° steps), covering three covariate conditions: normal walking (NM), walking with a coat (CL) and walking while carrying a bag (BG). Each subject performs six NM sequences, two CL sequences, and two BG sequences. CASIA-B was used in this study to evaluate the robustness of the proposed GSI representations under covariate variation. This includes the analysis of view changes and the impact of clothing and carrying conditions, which are known to significantly affect appearance-based gait representations.

### 3.2. Skeletal Topology

The construction of the *Gait Skeleton Image* requires selecting a skeletal topology that is at once discriminative for gait analysis, robust to pose-estimation inaccuracies, and computationally efficient. The adopted GSI representation employs a 17-keypoint body model designed to capture the most informative components of gait while avoiding joints prone to instability or with limited discriminative contribution.

Lower-body joints (hips, knees, ankles) form the core of the representation, as they exhibit substantial displacement and encode key gait parameters such as stride length, swing dynamics, and cadence. Upper-body joints (shoulders, elbows, wrists) provide complementary information through arm-swing coordination, which varies characteristically between individuals. Research consistently indicates that hand and foot extremities suffer higher tracking errors than joints closer to the body [17,18]. These extremities are therefore omitted, with feet and hands represented solely by ankle and wrist positions.

During lateral-view walking, the arm farthest from the camera is frequently occluded by the torso, leading to unstable or missing joint estimates and irregular template artifacts. Instead of reconstructing these joints, an approach that may introduce systematic errors, the GSI formulation relies exclusively on consistently visible keypoints. Facial landmarks are also excluded due to limited motion relevance and reduced detection reliability at gait-analysis distances. The resulting 17-keypoint model provides a balanced compromise between anatomical coverage and robustness, avoiding noisy extremities while retaining all gait-relevant motion.

To ensure consistency across pose-estimation frameworks, the GSI model defines a clear skeletal topology connecting the selected keypoints into a kinematic tree comprising the axial skeleton (head, neck, spine, pelvis) and the bilateral limbs. Connectivity follows natural anatomical chains and avoids cross-limb links, maintaining a hierarchical structure suitable for geometric normalization operations such as scale alignment and rotation compensation.

**Figure 2:**
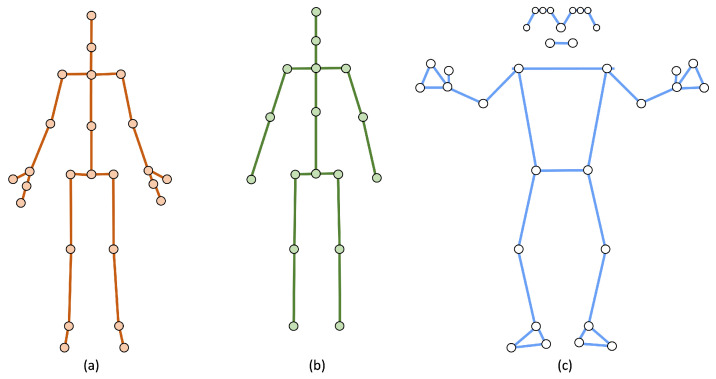
Comparison of body topologies. (**a**) Kinect v2: defined by 25 keypoints; (**b**) GSI: employs a selective 17-keypoint structure; (**c**) MediaPipe: includes 33 landmarks. The GSI topology retains only gait-relevant landmarks while excluding extremities and facial or hand landmarks.

The GSI pipeline supports multiple pose-estimation systems. MediaPipe and Kinect v2 both provide 3D joint coordinates but differ in anatomical coverage. MediaPipe outputs 33 landmarks, including facial and hand articulations beyond gait requirements; these are pruned, and four central-axis keypoints (neck, shoulder center, hip center, spine center) are computed via midpoint operations. Kinect v2 provides 25 joints more closely aligned with the GSI structure, allowing nearly all keypoints to map directly. The conversion protocol includes three categories: direct mappings, computed midpoints, and discarded non-gait landmarks. Figure 2 illustrates the 17-keypoint GSI topology compared with the original MediaPipe and Kinect v2 layouts, highlighting the anatomical simplifications and retained gait-relevant structures.

### 3.3. 3D Skeletal Coordinate Acquisition and 2D Normalized Projection

The construction of GSI representations requires reliable three-dimensional skeletal joint coordinates, which must be normalized and transformed into a canonical 2D reference frame prior to template generation. The three-dimensional joint coordinates can be obtained from depth-based sensors or monocular human pose estimation (HPE) applied to RGB imagery. Depth sensors typically provide direct metric joint positions, while RGB-based HPE infers them through learned depth estimation. Regardless of the acquisition method, the extracted skeletal representation is first converted to the unified 17-keypoint GSI topology using fixed mapping rules that retain only gait-relevant joints and discard or merge landmarks that contribute little to gait discrimination or suffer from reduced tracking reliability.

In order to guarantee viewpoint invariance, each three-dimensional skeleton is transformed through a sequence of rigid operations that align the body into a canonical lateral orientation. The procedure begins with a translation matrix T, which shifts the hip-center joint to the coordinate origin, followed by a rotation about the *Y*-axis, $R_{y}\left(\theta_{y}\right)$, that aligns the torso laterally with respect to the viewing direction. A conditional reflection matrix S is then applied whenever the body is oriented toward the negative *X*-axis, ensuring a consistent facing direction across all sequences. Finally, a rotation about the *Z*-axis, $R_{z}\left(\theta_{z}\right)$, corrects residual inclination induced by pose-estimation noise and stabilizes the torso within the XY-plane.

These operations follow standard homogeneous-coordinate conventions and combine into a single view-normalization matrix, defined as

(1) $$M_{\mathrm{view}}=R_{z}\left(\theta_{z}\right)\;S\;R_{y}\left(\theta_{y}\right)\;T.$$

Each joint in homogeneous coordinates, $p={\left[x\;y\;z\;1\right]}^{⊤}$, is transformed by the view-normalization matrix according to

(2) $$p^{\prime }=M_{\mathrm{view}}\;p,\;$$

where $p^{\prime }$ denotes the corresponding pose-aligned coordinate.

Because $M_{\mathrm{view}}$ in (1) is composed exclusively of rigid transformations (and reflection when required), the mapping in (2) preserves all metric properties of the motion while ensuring that each skeleton is consistently oriented along the positive *X*-axis.

Because gait sequences naturally include global body displacement across the scene, the joint coordinates must be stabilized prior to the construction of the GSI. A translation-normalization step is therefore applied to anchor each frame to a fixed spatial reference, removing the global trajectory while preserving the intrinsic joint kinematics and periodicity of the gait cycle. This produces locomotion-invariant sequences that are consistent across walking trials and suitable for template generation.

Subsequent to viewpoint and translation normalization, the 3D skeleton undergoes conversion into a 2D representation through orthographic projection. The body is reoriented so that the walking direction aligns with the positive *X*-axis and the gait motion unfolds primarily within the XY-plane. Orthographic projection removes the depth component (*Z*) while maintaining metric consistency in the XY-plane, ensuring that proportional 3D joint relationships are retained in the resulting 2D coordinates.

Ultimately, the projected points are rescaled to a fixed spatial canvas of 80 pixels in width by 120 pixels in height. This spatial normalization standardizes the coordinate range across subjects and acquisition modalities, thus producing consistent inputs for subsequent GSI rendering and 2D CNN processing.

### 3.4. Proposed GSI Representations

Two variants of the *Gait Skeleton Image* were developed to encode skeletal motion in a compact spatial-temporal 2D format. Both variants follow principles inspired by silhouette-based descriptors, namely, the aggregation of frame-wise motion into structured pixel templates, while relying exclusively on skeletal information.

#### 3.4.1. Line-Based GSI

**Figure 3:**
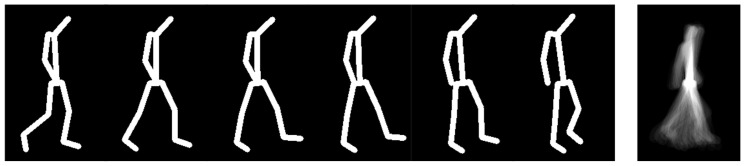
Example of the line-based GSI construction. Frame-wise skeletal contours are rendered using lines connecting anatomical joints; their temporal accumulation forms the final GSI template.

The line-based GSI encodes the skeletal topology by drawing lines between anatomically connected joints in each frame, using a specified line thickness. The resulting frame-wise contours are then temporally accumulated into a single 2D template that reflects both joint connectivity and the spatial-temporal envelope of body motion over the gait cycle. Examples of frames and the resulting template are shown in Figure 3. The influence of line thickness on smoothness and discriminative capacity was systematically evaluated using the GRIDDS dataset.

#### 3.4.2. Joint-Based GSI

**Figure 4:**
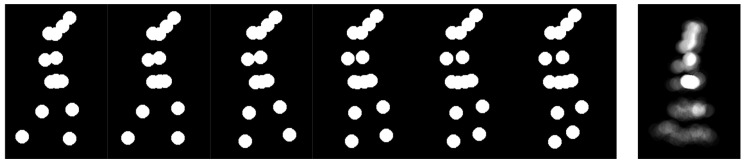
Example of the joint-based GSI construction. Circular markers of fixed radius are drawn at joint locations for each frame, and the temporal accumulation of these markers produces the final GSI template.

The joint-based GSI represents the skeleton by placing circular markers of radius *r* at each joint location. As with the line-based variant, these per-frame renderings are aggregated into a single static image, but this representation emphasizes individual joint trajectories rather than body-part connectivity. Figure 4 illustrates the construction process. Different marker radius sizes were tested to analyze the effects of spatial density, compactness, and expressiveness.

### 3.5. Rendering Parameter Selection

The rendering parameters of the GSI representations (line thickness in the line-based variant and circle radius in the joint-based variant) directly affect the spatial density, continuity, and discriminative strength of the resulting templates. Very small values produce sparse or fragmented contours that are more sensitive to pose-estimation noise, whereas excessively large values introduce blurring effects that obscure fine-grained kinematic details.

**Figure 5:**
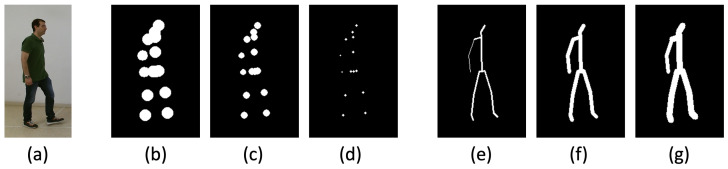
Visual examples of both GSI variants rendered with different pixel parameters. From left to right: (**a**) RGB reference image; (**b**–**d**) joint-based GSI with point radii r∈{2,8,12} pixels; (**e**–**g**) line-based GSI with line thickness values t∈{2,8,12} pixels. Larger radii or thickness values increase spatial density but may also smooth fine-grained kinematic details.

To determine suitable parameter values, both GSI variants were empirically evaluated using multiple configurations, with thickness and radius values set to {2, 4, 6, 8, 10, 12} pixels. A series of recognition experiments were conducted on the GRIDDS dataset, employing gait-cycle aggregated templates. Visual examples illustrating the effect of these pixel parameters on both GSI variants are presented in Figure 5. The experimental findings indicated that both variants achieved optimal performance with the 8-pixel configuration. Accordingly, all subsequent experiments adopt a line thickness of 8 pixels for the line-based GSI and a radius of 8 pixels for the joint-based GSI.

### 3.6. Unified Processing Pipeline

A unified pipeline was implemented for all experiments in both datasets. Each sequence was (i) loaded and temporally segmented, (ii) normalized in scale and coordinate space, (iii) converted to line-based or joint-based GSI format using the chosen pixel parameters, and (iv) resized to a fixed spatial resolution suitable for 2D CNN processing. The pipeline ensures that both GSI variants are generated under identical preprocessing assumptions, allowing a fair comparison of design choices.

### 3.7. CNN Architecture

The evaluation of both GSI representations and the GEI baseline was conducted using a deep 2D CNN model inspired by the GEINet architecture originally proposed by Shiraga et al. [19]. The network operates on single-channel input images and consists of two convolutional blocks followed by a fully connected classifier.

The architectural design was selected to closely follow the GEINet design, which is a well-established reference model in silhouette-based gait recognition. A detailed schematic representation and full architectural specification of GEINet are provided in the original work [19] and are therefore not reproduced here. Minor adaptations were introduced in the present implementation, namely the inclusion of batch normalization and dropout layers, to improve optimization stability and regularization without altering the overall model capacity.

Importantly, the same network configuration and training hyperparameters were used consistently for all input types (line-based GSI, joint-based GSI, and GEI). This design choice ensures that observed performance differences can be attributed to the properties of the input representations themselves, rather than to architectural advantages or differences in model complexity.

### 3.8. Training and Evaluation Protocol

In this study, two experimental settings were used, each with a distinct training and evaluation protocol. The first set of experiments, conducted on the GRIDDS dataset, aimed to analyze the design factors of the proposed GSI representations. The second set, conducted on CASIA-B, evaluated the recognition performance and robustness of the optimized GSI variants under covariate variation.

#### 3.8.1. Design-Factor Analysis (GRIDDS)

To examine the influence of pixel-level and structural encoding parameters, the GRIDDS experiments employed a leave-one-trial-out (LOTO) evaluation protocol. For each subject, trials 01–08 were used for training and trials 09–10 were reserved for testing. This setup ensures subject-independent identity recognition while enabling controlled comparison across rendering configurations.

Training was performed using the AdamW optimizer with an initial learning rate of $1\times 10^{-3}$, weight decay of $10^{-4}$, and a cosine learning-rate schedule with a 5-epoch warm-up. Early stopping with a patience of 10 epochs was used to prevent overfitting. The same CNN architecture was used for all parameter configurations, ensuring that performance differences arise solely from the GSI design choices. Classification accuracy on the held-out trials served as the evaluation metric.

#### 3.8.2. Robustness Under Covariate Variation (CASIA-B)

After selecting the optimal rendering parameters from the GRIDDS experiments, the two GSI variants were evaluated on the CASIA-B dataset to assess their robustness under three major covariates: viewpoint, clothing, and carrying conditions. The evaluation followed a standard CASIA-B protocol in which the first 74 subjects are used for training and the remaining 50 for testing. For each test subject, gallery templates were constructed from the first four normal walking sequences (NM#01–04), while probe samples comprised the remaining normal sequences (NM#05–06), the bag-carrying sequences (BG#01–02), and the coat-wearing sequences (CL#01–02).

To capture representative viewpoint variation while maintaining computational tractability, experiments were conducted on seven viewing angles: 0°, 36°, 72°, 90°, 108°, 144°, and 180°. This selection spans frontal, oblique, lateral, and back perspectives. Recognition was formulated as a closed-set identification task, and Rank-1 accuracy was calculated for each gallery–probe view pair, allowing a detailed analysis of both same-view and cross-view performance in all covariate conditions.

All models, GEI, GSI-Lines, and GSI-Joints, were trained under identical settings using cross-entropy loss and the Adam optimizer. This ensures a controlled comparison focused exclusively on representational differences, rather than architectural or optimization effects. Performance was reported separately for each viewpoint and covariate condition. This evaluation framework provides a direct and fair comparison between GEI and the two GSI variants and reveals how the proposed structural and pixel-level design choices impact recognition robustness, particularly in challenging cross-view and cross-condition scenarios.

## 4. Results

This section presents the experimental results obtained from the two complementary evaluation tasks: (i) rendering-parameter analysis on GRIDDS, and (ii) robustness assessment on CASIA-B under viewpoint, clothing, and carrying covariates. All results were obtained using the same CNN architecture and training setup described in Section 3, ensuring that performance differences arise exclusively from representational properties.

### 4.1. Rendering Parameter Evaluation

To determine the most effective rendering configuration for both GSI variants, a systematic parameter evaluation was conducted using the GRIDDS dataset under the leave-one-trial-out protocol. Six pixel-size values were originally designed for testing ({2,4,6,8,10,12}), and the GRIDDS recognition experiments revealed a consistent trend: performance increases as spatial support grows, reaching a peak at intermediate values before degrading with excessive smoothing.

Table 1 reports the Rank-1 identification rates for the best-performing configurations evaluated in GRIDDS. For joint-based GSI, the highest accuracy was achieved with a radius of 8 pixels (95.76%), followed by 12 pixels and 6 pixels. Similarly, the line-based GSI achieved its optimal performance with an 8-pixel line thickness (91.53%), with 10- and 4-pixel settings yielding slightly lower scores.

These results suggest that both excessively small radius/thickness values and excessively large ones compromise discriminative power. The intermediate value of 8 pixels was found to provide an optimal balance between density and structural clarity. Consequently, all subsequent CASIA-B experiments adopt: (i) a line thickness of 8 pixels for the line-based GSI; and (ii) a radius of 8 pixels for the joint-based GSI.

### 4.2. Recognition Under Viewpoint Variation

Table 2 and Table 3 present the full cross-view Rank-1 accuracies under normal walking conditions (NM gallery vs. NM probe) for the GSI-Joints and GEI representations, respectively, across frontal (0°), oblique (36°, 144°), lateral (72°, 90°, 108°), and back (180°) views.

Diagonal entries correspond to same-view recognition, while off-diagonals measure cross-view robustness. GEI demonstrates very high same-view performance, with diagonal entries close to saturation (mean around 96.9%), whereas GSI-Joints attains lower but still competitive same-view accuracy (mean around 74.8%). This confirms the strength of appearance-based silhouettes when the viewpoint is controlled.

When all view pairs (diagonal and off-diagonal) are averaged, GEI achieves an overall mean accuracy of 28.77%, and GSI-Joints achieves 26.98%. Considering only off-diagonal entries, i.e., strictly cross-view cases, both methods exhibit a pronounced drop in performance, and the mean cross-view accuracies become similar, with GSI-Joints slightly more stable under large viewpoint changes.

Under moderate angular differences between gallery and probe (e.g., 72° ↔ 90°, 90° ↔ 108°), GEI typically achieves higher recognition rates (often 70–85%), while GSI-Joints remain in the 45–58% range. However, for extreme viewpoint changes (e.g., 0° ↔ 90°, 90° ↔ 180°), GEI performance drops sharply and often approaches zero, while GSI-Joints, although still low, retains modest recognition rates (on the order of 8–11%). These results suggest that skeletal templates are less brittle under severe viewpoint changes, albeit at the cost of lower peak accuracy in favorable same-view configurations.

### 4.3. Recognition on Clothing and Carrying Conditions

Table 4 reports mean same-view accuracy across all seven viewpoints for the NM, BG and CL probe sets. GEI achieves the highest absolute accuracy but exhibits the greatest degradation under clothing variation (−25.7 percentage points), while both variants of GSI degrade less (approximately 20 percentage points), indicating an improved robustness to silhouette distortion.

The impact of carrying a bag (BG) is milder than that of clothing (CL) for all representations. GEI decreases from 96.9% (NM vs. NM) to 85.4% (NM vs. BG), while GSI-Lines and GSI-Joints drop from 73.0% and 74.8% to 67.2% and 68.9%, respectively. The smaller relative degradation observed for the GSI variants again suggests that skeletal encodings are less dependent on local appearance details, although GEI continues to achieve the highest absolute accuracy in same-view NM and BG conditions.

### 4.4. Comparison with GEI Baseline

Across all evaluations, GEI consistently demonstrates superior same-view accuracy, a consequence of its strong appearance encoding capabilities. However, the GSI variants demonstrate more gradual degradation under clothing and, to a lesser extent, carrying variation, and GSI-Joints exhibits slightly more stable behavior than GEI under extreme viewpoint changes. These trends indicate complementary strengths: appearance-based descriptors excel in favorable, well-aligned conditions, whereas skeleton-based GSIs offer enhanced robustness under appearance-altering and cross-view covariates.

## 5. Discussion

The experimental results demonstrate that the proposed *Gait Skeleton Image* (GSI) representations constitute a compact and interpretable alternative to traditional silhouette-based gait descriptors. Across all evaluated scenarios, the GSI variants consistently exhibit lower absolute Rank-1 accuracy than the Gait Energy Image (GEI) under favorable same-view conditions, particularly for normal walking. This behavior is expected, as GEI directly encodes appearance information that is highly discriminative when viewpoint and visual conditions are well controlled.

However, the results also reveal a complementary and practically relevant behavior. Under challenging covariate conditions, including viewpoint variation, clothing changes, and object carrying, the GSI representations exhibit more gradual and stable performance degradation than GEI. In particular, under extreme cross-view scenarios, GEI frequently suffers abrupt failures with near-zero recognition rates, whereas GSI retains modest but consistent discriminative capability. This indicates that skeletal motion cues preserve identity-related information even when appearance-based cues become unreliable or strongly perturbed.

From an application-oriented perspective, the choice between GEI and GSI should therefore be regarded as context-dependent rather than competitive. GEI remains the preferred representation in controlled environments where the camera viewpoint is fixed and appearance variations are limited, such as access-controlled indoor settings. In contrast, GSI becomes advantageous in scenarios where such assumptions cannot be guaranteed, including cross-view matching, outdoor surveillance, long-term monitoring, or situations involving clothing variability and carried objects. In these cases, the structural nature of skeleton-based representations offers increased robustness and avoids the abrupt performance drops observed for silhouette-based templates.

The rendering-parameter analysis conducted on the GRIDDS dataset further highlights the importance of careful pixel-level design in skeletal motion templates. Both line-based and joint-based GSI variants achieve optimal performance at intermediate spatial support values, with the 8-pixel configuration providing a balanced compromise between trajectory continuity and preservation of fine-grained kinematic detail. These findings emphasize that the discriminative capacity of skeleton-derived images is influenced not only by the underlying joint data, but also by how this information is spatially encoded and temporally accumulated.

From a computational perspective, the proposed GSI pipeline offers favorable trade-offs compared to alternative skeleton-based approaches. Once 3D joint coordinates are available, GSI construction involves only deterministic geometric normalization, orthographic projection, and image rendering operations, followed by standard 2D CNN inference. In contrast to graph-based or sequence-based skeletal methods, GSI avoids specialized graph convolutions, recurrent architectures, or multi-stream fusion, resulting in lower architectural complexity and easier deployment using widely optimized 2D CNN frameworks. While the overall runtime of the pipeline is dominated by the human pose estimation stage when RGB input is used, this cost is shared by all skeleton-based methods and is independent of the specific GSI formulation.

Beyond recognition accuracy alone, the use of compact 2D motion templates such as GSI offers practical and methodological advantages when compared to more complex deep or graph-based skeleton models. By converting variable-length skeletal sequences into fixed-size images, GSI enables the use of standard 2D CNN architectures with well-understood behavior, efficient implementations, and modest computational requirements. In contrast, graph-based or sequence-based models typically rely on specialized operations, temporal modeling, and increased architectural complexity, which may hinder reproducibility, deployment, and scalability in real-world settings.

Moreover, template-based representations provide an interpretable and visually meaningful abstraction of gait motion, allowing direct inspection of spatial and kinematic patterns that contribute to recognition performance. This interpretability is often lost in deeply stacked spatiotemporal or graph-convolutional models, where learned representations are harder to analyze and debug. As a result, simple 2D templates such as GSI strike a deliberate balance between representational expressiveness and computational simplicity, making them particularly attractive for applications where robustness, transparency, and ease of integration are as important as peak recognition accuracy.

The performance of GSI is intrinsically linked to the quality of the underlying human pose estimation (HPE). Since GSI relies exclusively on joint coordinates, inaccuracies in pose localization, temporal jitter, or missing keypoints directly affect the clarity and stability of the resulting templates. Nevertheless, recent advances in deep HPE have led to substantial improvements in 3D joint localization accuracy, robustness to occlusions, and temporal consistency. As these techniques continue to mature, skeleton-derived representations such as GSI are expected to benefit accordingly, yielding cleaner templates, enhanced discriminative capacity, and a progressively reduced performance gap relative to appearance-based baselines.

Overall, the results indicate that GSI provides a robust and interpretable complement to GEI rather than a direct replacement. While appearance-based descriptors remain dominant in ideal conditions, skeletal motion templates offer increased resilience under challenging covariates and a computationally efficient alternative aligned with recent advances in pose estimation. As such, GSI represents a promising direction for gait recognition systems operating in realistic, unconstrained environments, either as a standalone descriptor or as a component within future hybrid appearance–skeleton frameworks.

## 6. Future Work

Several directions can be pursued to further extend the present work.

First, while the use of GEINet provides a controlled and widely adopted baseline for representation-focused evaluation, future studies should consider stronger backbone architectures, including deeper CNNs and modern lightweight networks, to assess how the proposed GSI representations scale with increased model capacity. This includes architectures commonly used in recent gait-recognition literature as well as general-purpose image backbones.

Second, transfer learning constitutes a promising avenue for enhancing performance. Pretraining CNN backbones on large-scale image datasets or motion-related tasks, followed by fine-tuning on GSI templates, may allow the network to better exploit structural regularities and improve generalization, particularly under cross-view and appearance-changing conditions.

Third, hybrid evaluation protocols could be explored in which GSI representations are combined with appearance-based templates or other skeletal descriptors, enabling a more comprehensive assessment of complementary information sources under a unified learning framework.

In addition, future work will consider more extensive statistical analyses to further characterize performance variability and robustness. This includes the adoption of cross-validation strategies and multiple-run evaluation schemes, which would provide stronger statistical confidence in the reported results. Such analyses become particularly relevant as larger-scale datasets and increased computational resources become available.

Finally, as human pose estimation continues to improve in accuracy, temporal stability, and robustness to occlusion, future work should reassess the performance of GSI representations under these enhanced conditions. Improved pose quality is expected to yield cleaner and more discriminative templates, potentially narrowing the performance gap to appearance-based methods and further strengthening robustness in unconstrained scenarios. 

## Figures and Tables

**Table 1:** Parameter Optimization: Rank-1 Identification Rate on GRIDDS Dataset. Bold values indicate the best-performing configuration for each GSI variant.

Variant	Parameter	Rank-1 (%)
Joint-based	8 pixels	**95.76**
	12 pixels	93.64
	6 pixels	91.95
Line-based	8 pixels	**91.53**
	10 pixels	90.25
	4 pixels	90.68

**Table 2:** Cross-View Recognition Performance: GSI-Joints Rank-1 Rates (%) on the CASIA-B Dataset (NM Gallery vs. NM Probe). Bold values indicate the best recognition results, which occur in same-view conditions (diagonal entries), corresponding to matching gallery and probe viewpoints. Rows correspond to gallery views (↓) and columns to probe views (→).

Gallery ↓/Probe →	0°	36°	72°	90°	108°	144°	180°	Mean
**0°**	**75.26**	11.81	8.55	9.43	12.14	9.93	31.77	22.70
**36°**	18.53	**72.96**	23.44	10.26	16.63	18.33	4.85	23.57
**72°**	10.33	18.65	**74.21**	54.66	49.77	9.70	5.10	31.77
**90°**	10.89	8.14	57.78	**77.20**	50.42	8.61	9.16	31.74
**108°**	11.57	12.67	45.70	51.83	**74.48**	25.81	10.78	33.26
**144°**	9.61	26.92	8.45	7.20	28.47	**74.13**	13.08	24.01
**180°**	33.26	8.29	6.47	9.47	11.47	18.80	**74.96**	23.25
**Mean**	24.21	22.78	32.09	30.01	34.74	23.62	21.39	26.98

**Table 3:** Cross-View Recognition Performance: GEI Rank-1 Rates (%) on the CASIA-B Dataset (NM Gallery vs. NM Probe). Bold values indicate the best recognition results, which occur in same-view conditions (diagonal entries), corresponding to matching gallery and probe viewpoints. Rows correspond to gallery views (↓) and columns to probe views (→).

Gallery ↓/Probe →	0°	36°	72°	90°	108°	144°	180°	Mean
**0°**	**98.28**	6.10	0.47	0.16	1.10	3.60	31.77	20.21
**36°**	10.49	**96.40**	19.72	9.23	10.64	28.33	4.85	25.67
**72°**	2.50	15.65	**96.09**	84.66	49.77	9.70	1.10	37.07
**90°**	1.25	8.14	77.78	**97.18**	70.42	8.61	1.56	37.85
**108°**	1.72	7.67	50.70	71.83	**95.62**	15.81	0.78	34.88
**144°**	5.48	26.92	8.45	7.20	18.47	**96.40**	9.08	24.57
**180°**	34.12	3.29	0.47	0.47	0.47	10.80	**98.28**	21.13
**Mean**	21.98	23.45	36.24	38.68	35.21	24.75	21.06	28.77

**Table 4:** Same-View Recognition Under Carrying and Clothing Covariates on CASIA-B Dataset (Mean Across All Views).

Method	NM vs. NM	NM vs. BG	NM vs. CL	Mean
GSI-Lines	73.0%	67.2%	52.8%	64.3%
GSI-Joints	74.8%	68.9%	54.1%	65.9%
GEI	96.9%	85.4%	71.2%	84.5%
Degradation from NM:				
GSI-Lines	—	−5.8%	−20.2%	—
GSI-Joints	—	−5.9%	−20.7%	—
GEI	—	−11.5%	−25.7%	—

## Data Availability

The data presented in this study are openly available in CASIA-B at http://www.cbsr.ia.ac.cn/english/Gait%20Databases.asp (accessed on 4 January 2026).
